# Heat Waves and Morbidity: Current Knowledge and Further Direction-A Comprehensive Literature Review

**DOI:** 10.3390/ijerph120505256

**Published:** 2015-05-18

**Authors:** Mengmeng Li, Shaohua Gu, Peng Bi, Jun Yang, Qiyong Liu

**Affiliations:** 1 State Key Laboratory for Infectious Disease Prevention and Control, Collaborative Innovation Center for Diagnosis and Treatment of Infectious Diseases, National Institute for Communicable Disease Control and Prevention, Chinese Center for Disease Control and Prevention, Beijing 102206, China; E-Mails: limm55@126.com (M.L.); gushaohua1989@126.com (S.G.); smart_yjun@163.com (J.Y.); 2 Department of Epidemiology and Biostatistics, Institute of Basic Medical Sciences, Chinese Academy of Medical Sciences; School of Basic Medicine, Peking Union Medical College, Beijing 100005, China; 3 Climate Change and Health Center, Shandong University, Jinan 250012, China; 4 Discipline of Public Health, School of Population Health, The University of Adelaide, Adelaide 5005, Australia; E-Mail: peng.bi@adelaide.edu.au

**Keywords:** heat waves, morbidity, emergency medical care, hospitalization

## Abstract

In the past few decades, several devastating heat wave events have significantly challenged public health. As these events are projected to increase in both severity and frequency in the future, it is important to assess the relationship between heat waves and the health indicators that can be used in the early warning systems to guide the public health response. Yet there is a knowledge gap in the impact of heat waves on morbidity. In this study, a comprehensive review was conducted to assess the relationship between heat waves and different morbidity indicators, and to identify the vulnerable populations. The PubMed and ScienceDirect database were used to retrieve published literature in English from 1985 to 2014 on the relationship between heat waves and morbidity, and the following MeSH terms and keywords were used: heat wave, heat wave, morbidity, hospital admission, hospitalization, emergency call, emergency medical services, and outpatient visit. Thirty-three studies were included in the final analysis. Most studies found a short-term negative health impact of heat waves on morbidity. The elderly, children, and males were more vulnerable during heat waves, and the medical care demand increased for those with existing chronic diseases. Some social factors, such as lower socioeconomic status, can contribute to heat-susceptibility. In terms of study methods and heat wave definitions, there remain inconsistencies and uncertainties. Relevant policies and guidelines need to be developed to protect vulnerable populations. Morbidity indicators should be adopted in heat wave early warning systems in order to guide the effective implementation of public health actions.

## 1. Introduction

According to the Fifth Assessment Report (AR5) of the Intergovernmental Panel on Climate Change (IPCC) [1], the average global temperature from 2003 to 2012 was 0.78 °C higher than it was from 1850 to 1900. Global warming is projected to increase the frequency, intensity and duration of temperature extremes (e.g., heat waves and cold spells) under different Representative Concentration Pathway (RCP) scenarios both in the near- and in the long-term [2]. The short-term effect of heat waves, a prolonged period of excessively hot weather, on mortality has been well documented [3,4,5]. In the past few decades, several devastating heat waves have captured public health attention. For example, the 2003 France and the 1995 Chicago heat wave caused 15,000 and 700 excess deaths, respectively [6,7]. As the severity and frequency of heat waves are projected to increase [8], heat waves will pose even more challenges to our healthcare systems. Therefore, comprehensive understanding of its health impacts could provide evidence for early warning systems and adaptation to heat waves. Heat waves are not only associated with excess mortality, but also increase in morbidity. Since morbidity occurs before mortality, it might be more easily prevented by raising public awareness of heat waves and promoting early access to health care.

There has been increasing interest in the relationship between heat waves and morbidity. Various indicators have been used in this assessment, including the number of hospital admissions, outpatient department visits, emergency department (ED) visits, emergency hospital admissions (EHA), and ambulance call-outs [3,5,9]. However, the published studies have so far detected inconsistent results. These discrepancies may be associated with different regions, climates and demographic characteristics of the study sites as well as the various study designs applied. Therefore, it is necessary to review the updated studies to gain a comprehensive overview. The aims of this review are: (1) to understand the health impact of heat waves on population morbidity; (2) to assess the state of and gaps in current knowledge; (3) to comment on current research methodology; (4) to make evidence-based recommendation for public heath intervention programs; and (5) to highlight or pinpoint the direction of future research.

## 2. Methods

### 2.1. Search Strategy and Selection Criteria

The PubMed and ScienceDirect database were used to retrieve literature regarding the relationship between heat waves and morbidity. The primary search strategy followed U.S. National Library of Medicine’s Medical Subject Headings (MeSH) terms and keywords: heat wave OR heatwave AND morbidity OR hospital admission OR hospitalization OR emergency call OR emergency medical services OR outpatient department visit. We limited the search to original studies published in English between 1 January 1985 and 30 November 2014.

### 2.2. Eligibility Criteria

Studies that met the following eligibility criteria were included.

1. It is an original study.
2. Used heat wave as the main exposure of interest and included a clear definition of heat wave.
3. Used morbidity as the main health outcome. When morbidity and mortality are analyzed simultaneously, only the results on morbidity are included.
4. Studied the exposure-response relationship between heat waves and morbidity.

The studies that only used high daily temperature as exposure measures or did not have quantified effect estimates were excluded. Titles and abstracts were first screened, and then the remaining full articles were evaluated in detail to check whether they met the inclusion criteria. Further eligible articles were found through tracing the references of the articles generated by the primary search.

## 3. Results

**Figure 1:**
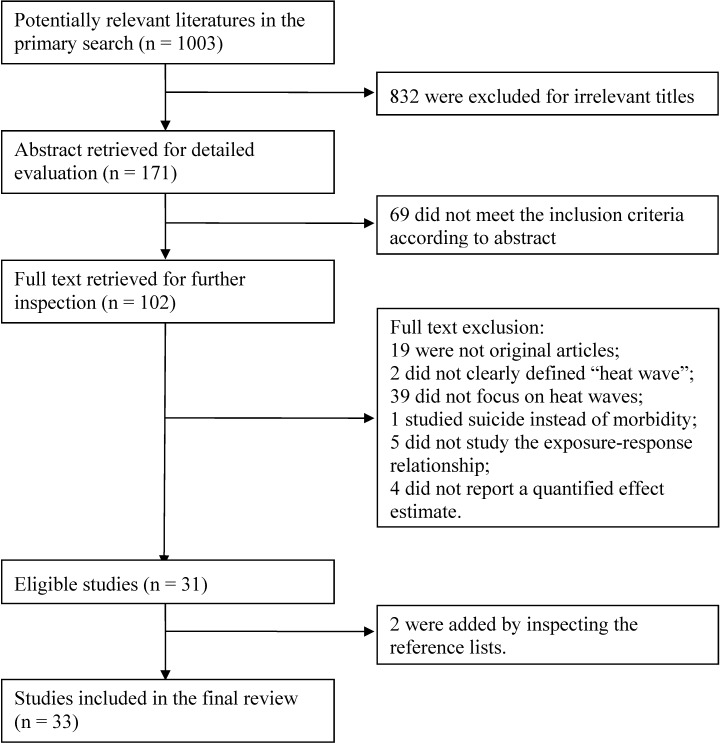
Literature searching process.

We identified 1003 papers in the initial search; 33 studies met the inclusion criteria and were included in the final review (Figure 1). Among them, 14 examined the relationship between heat waves and emergency medical care, eight investigated the impact of heat waves on hospital admissions, five assessed the emergency medical care and hospitalizations simultaneously, and six analyzed other data sources. Those studies were conducted in various regions, including nine in Europe, seven in the United States of America, 14 in Australia, two in China, and one in Canada.

### 3.1. Health Indicators

The heat wave-morbidity relationship can be measured by different indicators. Most of the current literature used hospitalization and emergency medical care related indicators, the latter of which can be further divided into ambulance call-out, emergency medical service (EMS) use, emergency department (ED) visit, and emergency hospital admissions.

Table 1 summarized the impact of heat waves on emergency medical care. As the front line of emergency medical service, ambulance services are greatly affected by emergency events. Existing studies consistently proved an increased demand for ambulance services during heat waves. For example, Turner [10] found an excess ambulance attendance of 18.8% during heat waves in Australia. Moreover, most studies detected that emergency department (ED) visits and emergency hospital admissions (EHA) increased during heat wave periods.

**Table 1:** Studies of the relationship between heat wave and emergency medical care.

Reference	Region and Time	Heat Wave Definition	Method	Outcome Variable	Key Findings	Effect Estimate (95% CI)	Comments
(Turner *et al.* 2013) [10]	Brisbane, Australia. 1 January 2000–31 December 2007	Greater than two consecutive days with daily Tmax ≥37 °C	Time-series; DLNM	Ambulance attendance for total, CVD and RD	Significant added heat wave effects were observed for both RD and CVD attendance, particularly for those aged 65–74 years (yrs).	CVD aged 65–74 yrs: percentage increase: 163.7% (56.0%, 345.8%);	The added effect of heat wave was evaluated after controlling for effect of main temperature and its lag by DLNM. Relative humidity, O_3_, NO_2_ and PM_10_ were also included.
						RD aged 65–74 yrs: percentage increase: 127.3% (14.7%, 350.3%).	
(Wang *et al.* 2012) [11]	Brisbane, Australia. 1 January 1996–31 December 2005	Greater than two consecutive days with daily Tmax ≥37 °C	Time-stratified case-crossover analysis	EHAs for CVD, RD, diabetes, ischemic stroke, Mental health and Renal disease	During heat waves, significant increases were observed in NEC aged 65–74 and ≥75 yrs and renal disease patients aged ≥75 yrs.	NEC aged 65–74 yrs: OR: 1.24 (1.02, 1.50);	Linear effects of relative humidity and air pollutants (PM_10_, NO_2_ and O_3_) were included.
						NEC aged 75+ yrs: OR: 1.39 (1.23, 1.58);	
						Renal patients aged 75+ yrs: OR: 2.25 (1.05, 4.83).	
(Tong *et al.* 2012) [12]	Brisbane, Australia. 1 January 1996–31 December 2005	Greater than two consecutive days with daily Tmax ≥37 °C	Time-series; Poisson GAM; time-stratified case-crossover analysis	EHAs	Heat waves were significantly associated with EHAs increase for both time-series and case-crossover method. The risk estimates gradually attenuated after the lag of one day.	Case-crossover analysis: OR: 1.22 (1.14, 1.30) at lag 1;	Comparison of both case-crossover analysis and time-series GAM model. Relative humidity, O_3_ and PM_10_ were included.
						GAM: RRs ranged from 1.14 (1.06, 1.23) to 1.28 (1.21, 1.36) at lag 1.	
(Schaffer *et al.* 2012) [13]	New South Wales, Sydney, Australia. 1 November–28 February for years 2006/2007 through to 2010/2011	30 January–6 February 2011	Time-series; Poisson regression model	Ambulance calls and ED visits	Significant increases in all-cause ambulance calls and ED visits were related with heat wave. And those aged ≥75 yrs had the highest impacts.	ED visits: RR: 1.02 (1.01, 1.03);	Heat wave was identified by the Bureau of Meteorology, Australia’s national weather agency. Day of the week, public holidays and quadratic variables for day of the year were included.
						Ambulance calls: RR: 1.14 (1.11, 1.16). Age, 75+ yrs:	
						ED visits: RR: 1.08 (1.04, 1.11);	
						Ambulance calls: RR: 1.17 (1.12, 1.23).	
(Mayner *et al.* 2010) [14]	Adelaide, Australia. 2009	The Bureau of Meteorology criterion: ≥5 consecutive days with Tmax ≥ 35 °C or ≥3 consecutive days with Tmax ≥ 40 °C	Kruskal-Wallis test (to check the differences between heat wave period and pre and post heat wave period)	ED patient presentation	Heat wave contributed to significant increases in number of total ED patient presentation compared to pre and post heat wave period.	Heat wave one: 18% and 9% increase in the number of ED patient presentations than pre and post heat wave period (*p* < 0.001);	A heat wave period was described by the Bureau of Meteorology. Demographic information and diagnostic descriptors were also presented.
						Heat wave two: 8% more than post heat wave period (*p* < 0.001).	
(Bustinza *et al.* 2013) [15]	Eight health regions of Quebec, Canada. 2005–2010	The days in July 2010 when the moving averages (over three days) of the Tmax and Tmin of the health regions were equal to or exceeded certain predefined thresholds	The normal approximation of the natural logarithm of the rate was used to calculate 95% CI of the crude rate	Emergency department admission rates	During heat wave, a relative small increase in emergency department admission was observed, compared to reference periods.	Percentage increase: 4% (*p* < 0.05).	Temperature threshold in each eight health region was used to define heat wave.
(Tong *et al.* 2010) [16]	Brisbane, Australia. January 1996–December 2005	Ten definitions	Case-crossover analyses	EHAs	During heat waves, there was a statistically significant increase in EHAs for all ten definitions.	OR ranged from 1.03 (1.01, 1.06) to 1.18 (1.11, 1.25).	Even a small change in the heat wave definition had an appreciable effect on estimated health impact. Relative humidity, PM_10_, NO_2_ and O_3_ were included.
(Bulbena *et al.* 2009) [17]	Barcelona, Spain. 2003	2–15 August 2003	Pearsons Chi Squared test, the Student’s T test and ANOVA)	Psychiatric emergency department	No differences were found in the number of emergencies and admissions, but significant differences were observed on alcohol and anxiety disorders comparing heat wave with non-heat wave days	Alcohol abuse disorders: OR: 2.12 (1.12, 4.01);	Data were from a general hospital and a psychiatric hospital.
						Anxiety disorders: OR: 0.47 (0.25, 0.89).	
(Cerutti *et al.* 2006) [18]	Ticino, Switzerland. 2001–2003	AT over 24 °C during three days or more, without falling below this threshold for more than one day, including the three days following the end of the wave	Data from 2001 to 2002 was used to estimate the mean of a Poisson distribution and 95% CI for the 2003 result, then calculated ratio O/E	Ambulance service intervention	The heat waves (especially in June) were correlated with a higher number of ambulance callouts.	Age, 65+ yrs: Ratio O/E: 1.21 (*p* < 0.05);	Three heat waves (*i.e.*, 8–30 June, 8–26 July and 2–20 August) were analyzed.
						Age, 75+ yrs: Ratio O/E: 1.21 (*p* < 0.05).	
(Johnson *et al.* 2005) [19]	Nine Government Office Regions in England. July–August 1998–2002	4–13 August 2003	Excess admissions were calculated as observed numbers minus the baseline (average of 1998 to 2002) expected number. Poisson distribution was used to calculate CI	Emergency hospital admissions	During heat wave, significant increases were found for 0–64 yrs and ≥75 yrs groups in London.	Percentage increase: Age, 0–64 yrs: 4% (1%, 6%); Age, 75+ yrs: 16% (12%, 20%).	The data covered nine Government Office Regions.
(Kovats *et al.* 2004) [20]	London, UK. 1 April 1994–31 March 2000	29 July–3 August 1995	Time-series; Poisson regression	Emergency hospital admissions	No obvious excess was apparent in the emergency hospital admissions.	Excess rate: 2.6% (−2.2%, 7.6%)	Long term trend, season, day of the week, public holidays, the Christmas period, influenza, relative humidity, air pollution (O_3_, PM_10_), and over-dispersion were controlled for.
(Leonardi *et al.* 2006) [21]	England 19 December 2001–23 May 2004	Two severe heat periods were defined as 10–19 July (10 days) and 4–13 August 2003 (10 days)	Time-series; GLM	Calls to NHS Direct(a nurse-led helpline)	Significant effect estimates were observed in July heat wave among children aged 0–4 yrs and 5–14 yrs.	Age, 0–4 yrs: 8.4 % (0.8%, 16.0%);	Data on calls to NHS Direct System was used. Long-term trend, day of the week and holiday, relative humidity, O_3_ and PM_10_ were included.
						Age, 5–14 yrs: 10.0 % (2.3%, 17.7%).	
(Gronlund *et al*. 2014) [22]	200 counties, USA. 1992–2006	Two-day mean AT above the 95th percentile of city-specific warm-season AT for 2–8 days in duration	Time-stratified case-crossover design with DLNM	Emergency hospital admissions for individuals ≥65 yrs	An added heat-wave effect with 8 days and 4 days in duration was observed on renal patient and RD, respectively	Renal disease: 12.8% (1.8%, 25.0%);	200 counties with highest number of cardiovascular hospital admissions in 2004–2006 were used. The analyses were stratified by age and gender.
						RD: 2.1% (0.1%, 4.3%).	
(Wang *et al.* 2014) [23]	Brisbane, Australia. 1996–2005	Daily Tmax of at least 37 °C (top 0.5%) for two or more consecutive days	Time-stratified case-crossover study	EHAs for renal diseases in children	There was a significant increase in EHAs for renal disease in children during heat wave. And highest risk estimates were observed at lag 1.	OR: 2.08 (1.05, 4.09) at lag 0–2;	Relative humidity, PM_10_, O_3_ and NO_2_ were included.
						OR: 3.6 (1.4, 9.5) at lag 1.	
(Williams *et al*. 2012) [24]	Perth, Australia. 1 January 2002–30 April 2009	Greater than three consecutive days with Tmax ≥ 35°C	GEE; negative binomial regression	EDs for total, mental, renal, CVD, RD	Heat waves days were associated with significant increases in total and renal disease EDs.	Total: IRR: 1.034 (1.011, 1.057);	Day of the week, month, year, O_3_, NO_2_ and PM_2.5_ were included.
						Renal disease: IRR: 1.109 (1.043, 1.180).	
(Nitschke *et al.* 2011) [25]	Adelaide, Australia. July 1993–March 2009	Greater than three consecutive days with Tmax ≥35 °C (the 95th percentile for Tmax for the period 1993–2009)	Case-series analysis; Negative binomial regression	Ambulance call-outs	During heat waves, highest effect estimates were (1) ambulance call-outs of respiratory patients aged 5–14 yrs (2) Emergency department presentations of direct heat disease aged 15–64 yrs	(1) RR: 1.47(1.13, 1.39).	The analyses were stratified by age group (0–4, 5–14, 65–74, 75+ yrs) and causes.
						(2) RR: 2.99 (2.24, 3.99).	
(Knowlton *et al.* 2009) [26]	58 counties of California, USA. 8 July 2006–22 August 2006	15 July–1August 2006	RR: the ratio of the number of cases in the heat wave and reference period. Excess cases: the difference of the number of cases in the two period	ED visits	During heat wave, a significant increase was observed in all age groups; highest effect estimate was observed on heat-related illness.	Total: RR: 1.03 (1.02,1.04);	The analyses were stratified by gender, age, causes and race.
						Heat-related illnesses: RR: 6.30 (5.67, 7.01)	
(Nitschke *et al.* 2007) [27]	Adelaide, Australia. 1993–2006	Greater than three consecutive days with daily Tmax ≥35 °C	Case-series study	Ambulance transports	During heat waves, a significant increase was found on total ambulance transport. Reductions were observed in relation to cardiac, sports- and falls-related events.	Total Ambulance transport: percentage increase: 4% (1%, 7%).	This analysis included five age groups (0–4, 5–14, 15–64, 65–74, 75+ yrs) and causes.
(Lindstrom *et al.* 2013) [28]	Melbourne, Australia. 2007–2009	28 January–3 February 2009.	Poisson regression	Emergency department presentations	During heat wave, a significant increase was observed in ED presentations.	ED presentations: IRR: 1.15 (*p* < 0.01)	One hospital data was used.

Abbreviations: Tmax: maximum temperature; Tmin: minimum temperature; AT: apparent temperature; DLNM: distributed lag non-linear model; GAM: generalized additive model; GLM: generalized linear model; GEE: generalized estimating equations; CVD: cardiovascular disease; RD: respiratory disease; NEC: non-external causes; EHAs: emergency hospital admissions; ED: emergency department; OR: odds ratio; RR: relative ratio; O/E: Observed/Expected; IRR: incidence rate ratio; CI: confidence interval.

Table 2 listed the papers that examined the heat waves and hospitalization association. Inconsistent results were observed. The magnitude of effect estimates varied in the studies that reported a significant adverse effect of heat waves on hospitalization. For example, the increase of all-cause hospitalizations ranged from 2% to 11% [29,30]. This discrepancy may be due to difference in health care system, demography and climatic characteristics as well as data availability. The results presented in Table 3 used data sources other than EMS or hospitalization, such as specific disease surveillance data, to identify the relationship between heat waves and certain diseases. For example, using birth defects data from New York State Congenital Malformation Registry, Van Zutphen *et al.* found that exposure to high temperature and heat waves in the critical period (e.g., weeks 3–8 post-conception, during organogenesis) was significantly associated with an increased occurrence of congenital cataracts [31]. As a surveillance disease in China, heat-related illness is diagnosed according to uniform criteria across all surveillance cities and registered through a national surveillance system. A sharp increase of heat-related illness was detected in Bai’ s study [32].

### 3.2. Vulnerable Populations

#### 3.2.1. Age

Published studies indicated that the elderly and children were easily affected by heat waves. Studies of ED visits, ambulance calls and emergency hospitalization showed that people aged over 75 years were more susceptible to heat waves than the youth [11,13,19]. Studies of heat waves and hospital admissions showed a similar result, with the risk increasing with age [33,34]. The younger children, aged 0–4 yrs, suffered the most during the 2006 heat wave in California [26], and excess morbidity of the youth (<14 yrs) had also been observed during a 10-day heat wave in England [21]. The odds ratio (OR) of heat wave impact on EHAs reached as high as 3.6 (95% CI: 1.4–9.5) [23] for renal diseases in children (aged 0–14 yrs), which indicated that children were at a greater risk on certain health consequences.

#### 3.2.2. Gender

The impact of heat waves on specific diseases was modified by gender. For instance, effects of heat waves on the stroke hospitalization, out-of-hospital cardiac arrest, heat-related illness, work-related injuries and diseases were significantly higher among males than among females [32,33,35]. However, these effects on renal diseases were higher among females than males [36]. This may be due to different exposures, working characteristics, and personal behaviors.

**Table 2:** Studies of the relationship between heat wave and hospitalization.

Reference	Region and Time	Heat Wave Definition	Method	Outcome Variable	Key Findings	Effect Estimate	Comments
(Manser *et al.* 2013) [37]	Zurich, Switzerland. 1 January 2001–31 December 2005	Any period of six days with Tmax > 5 °C above Tmax (recommended by the World Meteorological Organization)	Time-series; Poisson regression	Hospital admissions for IBD, IG and NIIs	Heat wave was significant associated with increased risk of IBD and IG flares. The strongest heat wave effect was observed on IG at lag seven days.	IBD flares: percentage increase: 4.6% (1.6%, 7.4%);	Data on one hospital was used. Day of the week, public holidays as Sundays, long-term trends and yearly seasonal patterns were adjusted.
						IG flares: percentage increase: 4.7% (1.8%, 7.4%);	
						IG flares: percentage increase: 7.2% (4.6%, 9.7%) at lag 7.	
(Ha *et al.* 2014) [33]	Allegheny County, Pennsylvania. May–September 1994–2000	Greater than two consecutive days with AT > 95th percentile (26.1 °C) of all temperatures.	Time-stratified case-crossover analysis	Stroke hospitalization for ischemic and hemorrhage stroke	Heat wave at lag-2 day was significantly associated with an increased risk for stroke hospitalization. The effect estimates were more significant for ischemic stroke, men and subjects aged 80 yrs or more.	Stroke: OR: 1.173 (1.047, 1.315) at lag2;	This analysis was stratified by gender, race, age group and type of stroke.
						Ischemic stroke: OR: 1.145 (1.009, 1.299) at lag2;	
						Male: OR: 1.201 (1.008, 1.430) at lag2;	
						Age, 65–79 yrs: OR: 1.161 (1.001, 1.346) at lag 2;	
						Age, 80+ yrs: OR: 1.191 (1.003, 1.414) at lag 2.	
(Ma *et al.* 2011) [29]	Shanghai, China. 2005–2008	Greater than seven consecutive days with daily Tmax above 35.0 °C and daily average temperatures above 97th percentile during the study period	The difference in the numbers of hospital admission between heat wave and reference period was used to calculate excess hospital visits, RRs and 95% CI	Hospital admission	The heat wave was significant associated with increase of total, CVD and RD.	Total: RR: 1.02 (1.01, 1.04).	The data were from database of Shanghai Health Insurance Bureau, covering most of the residents in Shanghai.
						CVD: RR: 1.08 (1.05, 1.11).	
						RD: RR: 1.06 (1.00, 1.11).	
(Hansen *et al.* 2008a) [36]	Adelaide, Australia. 1995–2006	Greater than three consecutive days when daily Tmax ≥35 °C, the 95th percentile of the Tmax range for the study period	Time-series; conditional-fixed effects Poisson regression;	Hospital admission for renal disease	During heat wave, significant increases were found on renal disease and acute renal failure. The effect estimates were higher among the elderly.	Renal disease: IRR: 1.100 (1.003, 1.206);	The analyses were stratified by three age group (15–64, 65+, 85+ yrs) and gender. Seasonality, long-term trend and over dispersion were controlled.
						ARF: IRR: 1.255 (1.037, 1.519).	
						Age,15–64 yrs: IRR: 1.130 (1.025, 1.247);	
						Age, 85+ yrs: IRR: 1.196 (1.036, 1.380).	
(Hansen *et al.* 2008b) [34]	Adelaide, Australia. 1 October–31 March 1993–2006	Greater than three consecutive days when daily Tmax ≥ 35 °C, the 95th percentile of the Tmax range for the study period	Time-series; conditional-fixed effects Poisson regression;	Hospital admission for MBDs	During heat wave, significant increases were found on patients with organic illnesses, including symptomatic mental disorders; dementia; mood (affective) disorders; neurotic, stress related, and somatoform disorders; disorders of psychological development; and senility. Higher effect estimates were observed on patients with senility.	MBDs: IRR: 1.073 (1.017, 1.132);	The analyses were stratified by three age group (15–64, 65–74, 75+ yrs), and gender. Seasonality, long-term trend and over dispersion were controlled.
						Senility: IRR: 2.366 (1.200, 4.667).	
(Semenza *et al.* 1999) [30]	Cook County, Chicago. 1994–1995	13–19 July 1995	Excess admission: the weekly average (the expected number of admissions) was subtracted from the number of admissions recorded during the heat wave study period. 95% CI: a standard method based on the t-distribution	Hospital admissions	Significant excess increases were observed on patients with disorders of fluid, volume depletion, nephritis, acute renal failure, heat stroke, anhydrotic heat exhaustion, heat exhaustion, hypertensive disease, ischemic heart disease, cardiac dysrythmias, diseases of arteries, cerebrovascular disease, late effects of cerebrovascular disease, diabetes mellitus, noninsulin dependent diabetes (Type II).	Significant excess rate ranged from 19% (*p* = 0.019) for ischemic heart disease to 78,000% (*p* < 0.001) for heat stroke.	Cause-specific hospital admissions were analyzed.
(Mastrangelo *et al.* 2007) [38]	Veneto Region, Italy. 1 June–31 August 2002–2003	Greater than three consecutive days with Humidex above 40 °C	GEE	Hospital admissions for the elderly (≥75 yrs)	Heat wave duration increased the risk of hospital admissions for heat disease and RD.	Heat diseases: IRR: 1.16 (1.12, 1.20);	Humidex was used; heat wave characteristics were analyzed, such as duration, intensity and a dummy variable for days outside or inside a heat wave.
					RD: IRR: 1.05 (1.03, 1.07).	No correlation was found for fractures of femur or circulatory disease admissions	
(Sheridan *et al.* 2014) [39]	New York, USA. April–August 1991–2004	Three consecutive days of DT (Dry Moderate) or MT+ (Moist Tropical Plus) weather type	Time-series; DLM	Hospitalizations for heat-related disease, CVD, RD.	The strongest effect of heat wave was observed on heat-related illness. Heat-related hospital admissions have increased during the time, especially during the earlier days of heat events.	Heat-related disease: RR: 25.891 (20.300, 33.022) during 1991–2004;	The analysis included different time period (1991–1996 and 1997–2004) and seasons (spring and summer).
						RR: 25.46 (15.98, 40.57) during 1991–1996;	
						RR: 26.69 (20.19, 35.29) during 1997–2004.	
(Williams *et al*. 2012) [24]	Perth, Australia. 1 January 1980–1 July 2008	Greater than three consecutive days with Tmax ≥35 °C	GEE; negative binomial regression	Hospital admissions	Total hospital admissions decreased during heat wave days.	Hospital admissions: IRR: 0.905 (0.854, 0.958).	Twenty-eight years of data were used.
(Nitschke *et al.* 2011) [25]	Adelaide, Australia. July 1993–March 2009	Greater than three consecutive days with Tmax ≥35 °C (the 95th percentile of Tmax for the period 1993–2009)	Case-series analysis; Negative binomial regression	Hospital admissions for total, ischemic, mental, renal, RD and direct heat disease	During heat waves, highest effect estimates were found on direct heat disease during 2008 and 2009 heat wave.	2008 heat wave: RR: 2.64 (1.32, 5.20);	The analyses were stratified by age group (0–4, 5–14, 65–74, 75+ yrs).
						2009 heat wave: RR: 13.66 (8.80, 20.98).	
(Knowlton *et al*. 2009) [26]	Fifty-eight counties of California, USA. 8 July 2006–22 August 2006	15 July–1 August 2006	RR: the ratio of the number of cases in the heat wave and reference period. Excess cases: the difference of the number of cases in the two period	Hospitalizations	During heat wave, a significant increase was found only on (1) Electrolyte imbalance, (2) Nephritis and nephritic syndrome (3) Acute renal failure (4) Heat-related illnesses	(1) RR: 1.09(1.07, 1.11)	The analyses were stratified by gender, age, causes and race.
						(2) RR: 1.05(1.02, 1.07)	
						(3) RR: 1.11(1.08, 1.15)	
						(4) RR: 10.15(7.79, 13.43)	
(Nitschke *et al.* 2007) [27]	Adelaide, Australia. 1993–2006	Greater than three consecutive days with daily Tmax ≥35 °C	Case-series study	Hospital admissions for CVD, RD, mental and renal disease	Highest effect estimate was observed for renal patients aged 15–64 yrs.	IRR: 1.16 (1.04, 1.30)	This analysis included five age groups (0–4, 5–14, 15–64, 65–74, 75+ yrs).
(Lindstrom *et al.* 2013) [28]	Melbourne, Australia. 2007–2009	28 January–3 February 2009	Poisson regression	Hospital admissions, general medical admissions	During heat wave, a significant increase was observed in hospital admissions and general medical admissions.	(1) IRR: 1.11 (*p* < 0.05)	One hospital data was used.
						(2) IRR: 1.81 (*p* < 0.01)	

Abbreviations: Tmax: maximum temperature; AT: apparent temperature; IBD: inflammatory bowel disease; IG: infectious gastroenteritis; NIIs: noninfectious chronic intestinal inflammations; CVD: cardiovascular disease; RD: respiratory disease; MBDs: mental and behavioral disorders; GEE: Generalized Estimating Equation; DLM: distributed linear model; OR: odds ratio; RR: rate ratio; IRR: incidence rate ratio; CI: confidence interval.

**Table 3:** Studies using other data sources other than EMS or hospitalization.

Reference	Region and Time	Heat Wave Definition	Method	Outcome Variable	Key Findings	Effect Estimates	Comments
(Van Zutphen *et al*. 2012) [31]	New York, USA. June–August 1992–2006	Greater than three consecutive days with mean UAT above the 90th percentile	Case control study	Birth defects	Congenital cataracts were significantly associated with heat waves, while significant decrease was on gastroschisis. No statistically significant relationships were found among central nervous systems, CVD, craniofacial or genitourinary birth defect groups.	Congenital cataracts: OR: 1.97 (1.17, 3.32);	A population-based case-control study was performed. The analyses were stratified by causes.
						Gastroschisis: OR: 0.48 (0.28, 0.81).	
(Schifano *et al*. 2013) [40]	Rome, Italy. 1 January–31 December 2001–2010	Greater than two consecutive days with MAT above the monthly 90th percentile or the daily Tmin above the monthly 90th percentile and MAT above the median monthly value	Time-series; DLM; Poisson GAM	Preterm birth	A significant increase of preterm birth was associated with heat waves.	Percentage increase: 19% (7.91%, 31.69%).	The long-term trend, seasonality and holidays were adjusted.
(Empana *et al.* 2009) [35]	Paris, France. 1 January–21 December 2000–2005	1–14 August 2003	Poisson regression analysis (the same period in years 2000–2002 and 2004–2005 as reference)	Out-of-hospital cardiac arrest due to heart disease and of ST-segment elevation myocardial infarction (STEMI) aged >18 yrs	During heat wave, a significant relative rate was found on out-of-hospital cardiac arrests but not on myocardial infarctions comparing to reference period. This increase estimates were higher among males and those aged above 60 yrs.	Out of Hospital Cardiac Arrests: RR: 2.34 (1.60, 3.41);	The data was from a city mobile intensive care units (MICU) database. The analysis was adjusted for gender and age.
						Myocardial Infarctions: RR: 1.09 (0.58, 2.03).	
(Kent *et al.* 2014) [41]	Alabama, USA. May–September 1990–2010	Sixteen heat wave definitions	Time-stratified case-crossover	Preterm births	Effect of heat waves (first definition) defined as having at least two consecutive days with Tmean above the 98th percentile were much higher than that as at least two consecutive days with T mean above the 90th (second definition).	The first definition: ER: 32.4% (3.7%, 69.1%);	Sixteen heat wave definitions were performed according to previous studies.
						The second definition: ER: 3.7% (1.1%, 6.3%).	Effect estimates varied by heat wave definitions.
(Bai *et al.* 2014) [32]	Ningbo, China. 2011–2013	Greater than seven consecutive days with the Tmax >35 °C	Time-series; DLNM	Heat-related illness	The strongest cumulative effect of heat waves was on severe forms of illness. Males and all age groups were vulnerable to heat wave.	The strongest cumulative effect (Lag 0–5): RR: 10.69 (2.10, 54.44).	The data were collected from the national heat-related illness surveillance system. The analyses included age groups and gender.
(Xiang 2014) [42]	Adelaide, Australia. 1 October–31 March 2001–2010	Greater than three consecutive days with daily Tmax ≥ 35 °C	GEE	Workers’ compensation claim	For outdoor industries, daily claims increased significantly during heat waves. And male laborers, tradespersons aged ≥55 yrs, those employed in ‘agriculture, forestry and fishing’ and ‘electricity, gas and water’, occupational burns, wounds, lacerations, and amputations as well as heat illnesses were significantly associated with heat waves.	Outdoor industries: IRR: 1.06 (1.02, 1.10)	The analyses were stratified by gender, age, occupation and industry.

Abbreviation: UAT: universal apparent temperature; MAT: daily maximum apparent temperature; Tmax: maximum temperature; Tmin: minimum temperature; Tmean: mean temperature; CVD: cardiovascular disease; DLM: distributed lag model; DLNM: distributed lag non-linear model; GAM: generalized additive model; GEE: generalized estimating equation; OR: odds ratio; RR: relative risk.

### 3.3. The Susceptible Patients

The relationship between heat waves and specific diseases has been fully explored, which shows different patterns among published studies. There is a consistent result indicating that the demand for medical care increased for people with renal diseases and heat-related illness [11,17,24,25,26,27,30,34]. For cardiovascular disease (CVD), respiratory disease (RD) and mental illnesses, the results remain inconsistent. For instance, a significant increase of 8% [29], 6% [29] and 7.3% [34] was found on hospital admissions for CVD, RD and mental diseases, respectively; and a much higher effect estimate of 16.1% increase was observed on stroke hospitalization [33]. However, other studies detected non-significant association [11,14,24,26]. Negative health effects of heat waves have also been detected for other diseases such as preterm birth [31,41], birth defect [31], electronic imbalance [26], inflammatory bowel disease and infectious gastroenteritis [37]. However, conclusion cannot be reached due to lack of sufficient evidence, and further studies are needed.

### 3.4. The Additional Effect of Heat Waves on Morbidity

Heat wave, an episode of sustained heat, may have an extra effect on morbidity above and beyond that of high temperatures alone because of its intensity and duration. In order to estimate the additional effect of heat wave on morbidity, the general effects of high temperature would need to be removed. Comparing the observed rate of calls to National Health Service (NHS) Direct (a nurse-led helpline in England) to the expected values after controlling for general temperature effect, Leonardi *et al.* found that the total symptomatic calls were moderately elevated during the two heat episodes in 2003 [21]. Turner *et al.* discovered that heat wave accounted for 18.8% (95% CI: 6.5%, 32.5%) additional total ambulance attendance in Brisbane, Australia [10]. Similar effects were also observed for renal and respiratory admissions in the United States [22]. However, in a study of the 1995 heat wave in Greater London, Kovats *et al.* observed no excess morbidity due to heat wave. Although there was a slight increase in the additional effect due to heat wave on hospital admissions using the regression model without temperature terms in the sensitivity analysis, it hardly reached statistical significance [20]. Such inconsistent results might be due to differences in medical diagnostic criteria, demographic characteristics and climatic conditions. More studies are required to elucidate the additional effect of heat waves on morbidity.

### 3.5. The Lag Effect of Heat Waves

Most of the studies showed that heat waves had a short lagged effect limited within seven days on morbidity. The lag pattern of heat waves varied across regions and diseases. Wang *et al.* showed that exposure to heat at lag 0, lag 1, and lag 0–2 (three days’ cumulative exposure) significantly increased the risk of EHAs for children’s renal diseases in Brisbane, a sub-tropical city in Australia, and the strongest heat wave effects appeared at lag 1 [23]. A similar result was obtained by this research group when examining the lagged effect of heat waves on EHAs within a scope of five days [12]. Schaffer *et al*. found that the greatest relative risk of heat waves on all-cause ambulance calls and ED visits was at lags of one and three days in Sydney, respectively [13]. In another study, heat wave at a two-day lag was found to be significantly associated with the risk of hospitalization for stroke (OR 1.173, 95% CI: 1.047–1.315) [33]. However, the effect of heat waves on infectious gastroenteritis had a longer lag effect, the strongest of which was observed at lag day 7 [37].

## 4. Discussion

Heat waves are associated with a series of health problems ranging from increased morbidity to excess death toll. Numerous studies have examined the harmful impact of heat waves on mortality with nearly consistent results [3,43]. However, the number of studies on the heat wave-morbidity relationship is relatively small and their outcomes varied across regions. Heat wave-related morbidity could be a useful indicator to inform the early warning system and adaptive measures. However, our review shows that knowledge gaps on the heat wave-morbidity association still exist.

### 4.1. The Impact of Heat Waves on Emergency Medical Care and Hospital Admissions

Most of the existing literature identified the negative effect of heat waves on emergency medical care. However, inconsistent results were found among studies on hospital admissions. This inconsistency might be due to various social factors that can veil the modest environmental effect. For example, the number of available beds is often limited, so most patients will be treated in the ED and then sent home. Only a small number of patients are hospitalized in each study location during heat waves, which may reduce the statistical power. Our search may be skewed towards positive results because of an inherent publication bias—the negative or statistically non-significant results may not be openly published. It might indeed be the case that the adverse effect of heat waves on hospital admission is a lot smaller than on emergency medical services use because ambulances and ED are really at the front line of medical assistance, and thus are more sensitive to acute events.

### 4.2. The Vulnerable Populations

The risk posed by heat waves is not of the same magnitude in different age and gender groups. The elderly, children and males are more susceptible than other subgroups. The elderly aged over 65 years old is found to be a vulnerable group in most studies, which is also in line with studies on the temperature-morbidity relationship [9]. The regulation of body temperature involves multiple organ systems, which naturally deteriorates with the aging process. This leaves the elderly more susceptible to heat [44], especially when high temperature sustains for several consecutive days, giving the body no time to regulate back to normal. Furthermore, the elderly with existing chronic diseases are at even higher risk, because physiological impairments may already weaken their ability to deal with sustained heat [45], and medications they take may contribute to further increase in this risk [36]. Besides the physical capability to deal with extreme weather events, the elderly could also face social isolation [46], which needs to be addressed for early warning and adaptation purposes. Josseran’s study showed that the elderly would require more vigilance and preventative efforts during heat waves [47], such as the establishment of an elderly contact database during heat waves. Children are more vulnerable to heat waves than adults because their regulatory systems are less developed. They have greater metabolic rates and lower cardiac output. In addition, they tend to spend more time outdoors and take no protective measures during heat waves [48]. Therefore relevant regulations need to be established for scheduled school sports activities during heat waves. The reason why men suffer more from heat waves may be partly due to the fact that they are more likely to participate in outdoor activities and therefore be exposed to heat in hot days. For example, Xiang found that male workers had more work-related injuries than females during heat waves [42]. Whether a biological mechanism lies beneath the gender gap still needs further research and investigation.

People with specific diseases are vulnerable to heat waves. Among the studies that explored those susceptible diseases, consistent evidences showed adverse health effects of heat waves on heat-related and renal diseases morbidity. Heat-related disease tend to occur during outdoor labor as a result of accumulated heat load over a longer time period with little opportunity for rest. Renal health can be compromised during extreme heat days due to dehydration and hyperthermia leading to electrolyte and water imbalance and placing extra stress on the kidneys [36]. However, the results on cardiovascular and respiratory morbidity varied from one study to another. Some studies speculated that the heat wave effect on these diseases may be partly accounted for by the air pollution effect during heat waves. Further epidemiological and pathogenetic researches are warranted to address this uncertainty.

Social factors can also affect people’s responses to heat waves. The socioeconomic status (SES) was an effect modifier between temperatures and emergency hospital admission in Wichmann’s study in Denmark [49], and those with low SES had increased morbidity and mortality during extreme heat [50]. As two important indicators of SES, Yang *et al.* [51] found that heat wave-associated mortality was modified by education level and occupation class. Xiang *et al.* [42] also reported that outdoor workers, in particular those undertaking highly intensive and physical activities were at high risk of heat-related illnesses and injuries during extremely hot weather. Moreover, according to Hansen’s study, people living in culturally and linguistically diverse (CALD) communities were also vulnerable to heat [52]. A possible explanation may be that they are not able to get the necessary information and resources and their living and housing conditions may be more disadvantaged, which could worsen their ability to cope with heat waves [53]. It is therefore important to work together with relevant government organizations, non-governmental organizations (NGOs) and service providers for appropriate and relevant health interventions.

### 4.3. The Heterogeneity of Existing Literature

The effect estimates of different studies cannot yet be synthesized to obtain a pooled result because of the challenges of inconsistent methodology and heat wave definition. Typically, the most common statistical approach in this field is time-series analysis with Poisson regression to obtain the relative risk. Some other studies calculated excess morbidity by directly comparing the observed numbers of events during heat waves and the expected numbers of events based on the reference period [14,17,19,22,26,29,30]. The reliability of the results can be influenced by various choices of the baseline period. Case-crossover design is another common method to explore the relationship between heat waves and morbidity by calculating the odds ratio and confidential intervals. Given the methodological heterogeneity in the current available literature, it is important to evaluate different methods in order to assess the most appropriate method in detecting heat wave impact on morbidity and to compare studies conducted in different regions of the world.

The current studies used various heat wave definitions, which were usually based on local meteorological conditions. In the studies that compared different definitions, Turner *et al.* found that the effects of heat waves generally increase with higher percentiles of maximum temperature used in the definition [10]; Tong *et al.* assessed the relationship between 10 definitions and emergency hospital admissions and found that even a small change in the heat wave definition produced an appreciable effect on the estimated health impact, with odds ratios ranging from 1.03 to 1.18 [16]. In addition to temperature, other meteorological factors, such as relative humidity, rainfall and wind velocity, are also used to form a comprehensive indicator—apparent temperature and heat index, for example [41,54]. Although heat waves are usually locally defined and have no universal definition, duration, intensity and timing are their essential characteristics, which should be considered in data analysis. According to one study, high intensity, long duration and being the first heat wave of the summer are the factors that make heat waves more harmful [55]. However, the exact combination of these three characteristics may differ across regions. Therefore, more studies are needed to identify the contributions of the duration, intensity and order of heat waves. Recently, Excess Heat Factor (EHF) has been used in the study of heat and population health, which is a new heat index based on three-day-averaged daily mean temperature that efficiently captures the heat wave severity in Australia. Such comprehensive indicators should be further evaluated on their effectiveness in detecting heat waves [56]. Even when using the same definition, different heat waves may have different impact on morbidity [25]. All of the above-mentioned problems could pose difficulties to the pooling of effect estimates from the current literature.

### 4.4. Usage of Morbidity Data in the Heat Wave Early Warning System

Considering the health impact of heat waves, surveillance and early warning systems are needed to enable policy-makers and stakeholders to take immediate and effective actions. Heat wave and health warning systems based on weather forecast data did not seem to capture heat wave effect very well. In a study in Detroit, Michigan found that prediction of mortality from forecast heat waves tended to underestimate heat wave effects relative to the effect of observed weather metrics [57]. Therefore, other critical input should also be considered in activating heat alerts and warnings. Even for the observed meteorological data, studies have found that different weather variables and exposure metrics performed differently in predicting the health outcomes. The most important predictors should be identified and applied in the heat wave early warning system [58,59]. Besides meteorological predictors, surveillance of health indicators, including morbidity and mortality, should also play an essential role when creating heat wave response plans. Previously, surveillance systems based on mortality data have already been developed and implemented. For example, the Heat-Health Watch system established in England, Wales and Italy since 2004 as part of the national heat wave plan is a timely mortality surveillance system [60,61], the monitoring system of all-cause mortality in Belgium (Be-MOMO) is also a sensitive measure that can generate early warning [62]. The inclusion of morbidity-based health indicators can help to optimize the early warning system. For instance, the syndromic surveillance system based on emergency departments’ morbidity rates satisfactorily detected the health impact of hot days in France [63]. Since calls to NHS Direct are sensitive to daily temperatures and extreme weather, calls for heat- and sun-stroke are now routinely monitored as part of the UK Heat-wave plan [21]. The Montreal public health surveillance system monitored health indicators such as total mortality, pre-hospital emergency transports, and calls to the health information line and hospital admissions during the 2010 heat wave to guide the decision to implement medical interventions and actions [64]. In South Australia, Telecross REDi Service was activated in 2009 as a part of the State Heat and Health Emergency Response to provide calls to vulnerable clients during extreme heat days, especially the senior population. However, there has been no systematical way of evaluating these early warning systems to examine the effectiveness of such systems in reducing morbidity and mortality due to heat waves. This is an area that should be explored in future.

### 4.5. Limitations and Future Considerations

Current studies have provided a general overview of the association between heat waves and morbidity, but there are still a lot of knowledge gaps that need to be filled, such as the definitions of heat waves and the methodology used to best assess heat wave effect. Specifically, the heterogeneity in heat wave definition and methodology makes it difficult to synthesize the existing research to get a quantitative result. Different methods should be assessed to evaluate their relative ability to detect the impact of heat waves on morbidity, and the various definitions of heat waves should be applied to the local health data to find the most suitable one to guide local action, as there is not a universal definition because of the diversity in local weather and social conditions. Susceptible diseases and vulnerable populations require further exploration. Current strategies and plans should be evaluated to test their effectiveness. Approaches and measures taken should be evidence-based and apply to local condition.

Furthermore, most existing heat waves and morbidity studies are from developed countries such as Europe, Australia and the USA. There are very limited studies from developing countries such as China and India. It is important to examine the effect of heat waves on morbidity in these heavily populated countries and to establish the necessary comprehensive data collection and surveillance systems required.

## 5. Conclusions

Although inconsistency still remains in the literature, most studies found harmful health effects due to heat waves. The direction of these effects on emergency medical care is more consistent. Policy-makers and key stakeholders should make strategies to protect vulnerable populations, especially the elderly and children, people with lower SES, the CALD community, and people with chronic diseases. Morbidity indicators should be adopted in heat wave early warning systems to guide the implementation of effective public health actions. Multi-region study and even international cooperation using identical methodological strategy and various heat wave definitions are warranted to cope with current knowledge gaps on this important health concern.
